# Which is more generalizable, powerful and interpretable in meta-analyses, mean difference or standardized mean difference?

**DOI:** 10.1186/1471-2288-14-30

**Published:** 2014-02-21

**Authors:** Nozomi Takeshima, Takashi Sozu, Aran Tajika, Yusuke Ogawa, Yu Hayasaka, Toshiaki A Furukawa

**Affiliations:** 1 Department of Health Promotion and Human Behavior, Kyoto University Graduate School of Medicine/School of Public Health, Yoshida-Konoe-cho, Sakyo-ku, Kyoto 606-8501, Japan; 2 Department of Biostatistics, Kyoto University Graduate School of Medicine/School of Public Health, Yoshida-Konoe-cho, Sakyo-ku, Kyoto 606-8501, Japan; 3 Department of Clinical Epidemiology, Kyoto University Graduate School of Medicine/School of Public Health, Yoshida-Konoe-cho, Sakyo-ku Kyoto 606-8501,Japan

## Abstract

**Background:**

To examine empirically whether the mean difference (MD) or the standardised mean difference (SMD) is more generalizable and statistically powerful in meta-analyses of continuous outcomes when the same unit is used.

**Methods:**

From all the Cochrane Database (March 2013), we identified systematic reviews that combined 3 or more randomised controlled trials (RCT) using the same continuous outcome. Generalizability was assessed using the I-squared (I^2^) and the percentage agreement. The percentage agreement was calculated by comparing the MD or SMD of each RCT with the corresponding MD or SMD from the meta-analysis of all the other RCTs. The statistical power was estimated using Z-scores. Meta-analyses were conducted using both random-effects and fixed-effect models.

**Results:**

1068 meta-analyses were included. The I^2^ index was significantly smaller for the SMD than for the MD (*P* < 0.0001, sign test). For continuous outcomes, the current Cochrane reviews pooled some extremely heterogeneous results. When all these or less heterogeneous subsets of the reviews were examined, the SMD always showed a greater percentage agreement than the MD. When the I^2^ index was less than 30%, the percentage agreement was 55.3% for MD and 59.8% for SMD in the random-effects model and 53.0% and 59.8%, respectively, in the fixed effect model (both *P* < 0.0001, sign test). Although the Z-scores were larger for MD than for SMD, there were no differences in the percentage of statistical significance between MD and SMD in either model.

**Conclusions:**

The SMD was more generalizable than the MD. The MD had a greater statistical power than the SMD but did not result in material differences.

## Background

A meta-analysis aggregates indexes of effectiveness of individual trials into one pooled estimate. When the outcome of interest is a dichotomous variable, the commonly used effect sizes include the odds ratio (OR), the relative risk (RR), and the risk difference (RD). When the outcome is a continuous variable, then the effect size is commonly represented as either the mean difference (MD) or the standardised mean difference (SMD) [1].

The MD is the difference in the means of the treatment group and the control group, while the SMD is the MD divided by the standard deviation (SD), derived from either or both of the groups. Depending on how this SD is calculated, the SMD has several versions such, as Cohen's d [2], Glass's Δ [3], and Hedges' g [4].

When the outcome is measured in different units across trials, then we have no other choice but to use the SMD to combine the outcomes in the meta-analyses. On the other hand, when the outcome is measured in the same unit in every trial, theoretically, we can use either the MD or the SMD. In this latter case, there currently appears to be no unanimous agreement about which effect size is preferable, and different textbooks of meta-analyses provide differently nuanced recommendations about the selection of the appropriate effect size for continuous variables.

According to the Cochrane Handbook for Systematic Reviews of Interventions [1], the “selection of summary statistics for continuous data is principally determined by whether studies all report the outcome using the same scale when the MD can be used.” The American Psychological Association (APA) Task Force on Statistical Inference maintains that “if the units of measurement are meaningful on a practical level (e.g. number of cigarettes smoked per day), then we usually prefer a MD to a SMD” [5]. Egger et al. writes that “the overall treatment effect [in terms of SMD] can also be difficult to interpret as it is reported in units of standard deviation rather than in units of any of the measurement scales used in review” [6].

On the other hand, there are also authors who recommend the SMD along with or over the MD. The APA Publication Manual suggests that it can often be valuable to report not only the MD but also the SMD [7]. Borenstein, in his “Introduction to Meta-Analysis” [8], wrote that if the unit is unfamiliar, the SMD serves as an easy way to judge the magnitude of the effect, thanks to the general rules of thumb described by Cohen that suggest that an SMD of 0.2 represents a “small” effect, an SMD of 0.5 represents a “medium” effect, and an SMD of 0.8 represents a “large” effect [2]. For example, when you read that a treatment group’s mean post-treatment score on scale X was 10 points higher than that of a control group, there is no way of appreciating how much a difference this actually represents unless you are very familiar with the scale that is being used. But if the difference is expressed in terms of SMD as corresponding to an effect size of 0.5, for example, you can understand that it represents a moderate effectiveness in comparison with the control. In fact, Tian et al. noted that the SMD does not depend on the unit of measurement, and therefore the SMD has been widely used as a measure of intervention effect in many applied fields [9].

The preferability of the MD or the SMD can be examined from three aspects. First, which of the MD or the SMD is clinically more interpretable? The above-summarised arguments made by different authors seem to concern mainly this aspect. Second, which of the MD or the SMD is more generalizable (as any summary index should have a good generalizability so that it can be applied to the next group of patients) [10]? Third, which of the MD or the SMD is statistically more powerful (as we expect meta-analyses to provide as precise an estimate of the treatment effect as possible and to be as sensitive as possible to differences among treatments)?

To the best of our knowledge, no systematic assessment focusing on the second or third aspects of the MD and the SMD has been conducted. The objective of this research was, therefore, to examine empirically which index is more generalizable and statistically powerful in meta-analyses when the same unit is used: MD or SMD?

## Methods

### Selection of meta-analyses

We included the following intervention meta-analyses from the Cochrane Database of Systematic Reviews (March 31, 2013) [11,12].

1. The outcome of the meta-analyses was a continuous outcome in the same unit.

2. There were at least three studies contributing to that continuous outcome in question, because we need at least three studies in order to calculate the percentage agreement that we defined as one of our outcome (see below).

3. If there were two or more outcomes that met the above criteria, we selected the outcome that contained the greatest number of studies in the review. If there were 2 or more such outcomes, we chose the outcome reported for the greatest number of patients. If the numbers of the patients were the same, we selected the outcome that appeared first in the review.

We excluded the outcomes reported in sensitivity analyses or subgroup analyses.

### Search methods and data extraction

We searched the Cochrane Database of Systematic Reviews (March 31, 2013) using the term “mean difference” in all fields to identify all meta-analyses possibly meeting the above eligibility criteria.

One author examined all the meta-analyses identified using the above search methods to decide whether the meta-analysis met our inclusion criteria and chose which outcome to focus on. In order to evaluate the reliability of the selections, we randomly selected 100 out of the original set of meta-analyses and the other authors examined the reliability of these selections. We then calculated the kappas for these selections.

### Outcomes

To look at generalizability, we examined (i) the I-squared (I^2^) index and (ii) the percentage agreement. To compare statistical power, we examined (iii) the Z-score.

(i) I^2^ index

The I^2^ index represents the degree of heterogeneity across studies in meta-analyses [13]. It ranges from 0% to 100% and the following rough rule of thumb has been proposed [1]:

0% to 40%: might not be important

30% to 60%: may represent moderate heterogeneity

50% to 90%: may represent substantial heterogeneity

75% to 100%: considerable heterogeneity.

Because more heterogeneous results are expected to show less generalizability to similar populations, we examined the I^2^ index of each meta-analysed MD and SMD. We used the sign test to compare the I^2^ values for the MD and the SMD.

(ii) Percentage agreement

One study was extracted from each meta-analysis. The MD and the SMD of that individual study were then compared with the meta-analytically pooled MD and the SMD of the remaining studies. Agreement was defined when the point estimate of the MD or the SMD of the individual study was included within the 95% confidence interval of the pooled MD or SMD of the remaining studies [10]. This procedure was repeated for all the studies, and the overall percentage agreement was calculated. We calculated two percentage agreement figures for each meta-analysis, using the random-effects model [14] or the fixed-effect model [15] for the meta-analysis. In each model, we compared the percentage agreement of the MD with that of the SMD using the sign test.

(iii) Z-score

We also examined the Z-score or each meta-analysis in order to examine possible differences in the statistical power between the MD and the SMD using the sign test. In addition, we compared the MD or SMD values that were judged to be statistically significant at a conventional level (*P* < 0.05) using the McNemar test.

In order to calculate the percentage agreements, the I^2^ statistics, and the *P*-value for the treatment effect between the MD and the SMD, we used the inverse variance method meta-analyses that are included in SAS, Ver. 9.3 (SAS Institute Inc.). We defined SMD as Hedges’ adjusted *g* to remove any upward bias that might have arisen because of small sample sizes [16].

## Results

### Search results

**Figure 1:**
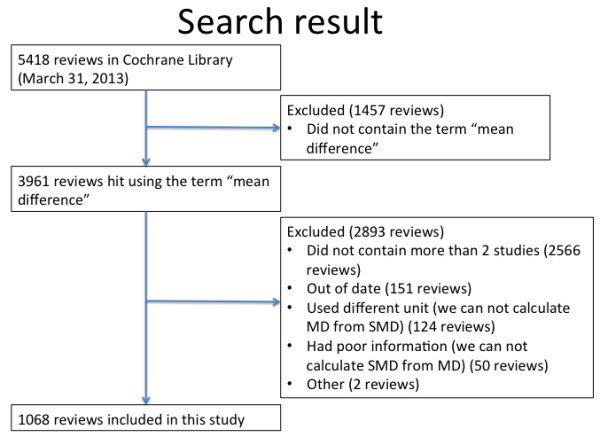
Flowchart for selection of meta-analysis.

Figure 1 describes the process of our search. There were 5418 reviews in the Cochrane Database of Systematic Reviews (March 31, 2013). When we searched the database using the term “mean difference” in all the texts, 3961 reviews were selected. Of these, 1068 reviews met our eligibility criteria. The main reason why the reviews were excluded was that the outcome of the meta-analyses contained two or fewer studies. Among the 1068 meta-analyses in our dataset, only 47 (4.4%) meta-analyses reported SMD, and 1021 (95.6%) meta-analyses reported MD. In order to confirm the reliability of this identification, two authors independently examined 100 reviews that were randomly selected from the 3961 reviews. The kappa was 0.90.

The median number of the studies contributing to each outcome was 5 (interquartile range: 3 to 8). The median total of participants used to determine the outcome was 489 (interquartile range: 218 to 1146). The median absolute SMDs for the random-effects model and for the fixed-effect model were 0.29 (interquartile range: 0.12 to 0.56) and 0.32 (interquartile range: 0.14 to 0.62), respectively. The subject areas of the 1068 reviews were as follows: internal medicine, n = 404; obstetrics and gynaecology (including paediatrics), n = 234; surgery, n = 204; psychiatry, n = 140; anaesthesiology, n = 33; and others, n = 53.

### Outcomes

#### I^2^ index

**Figure 2:**
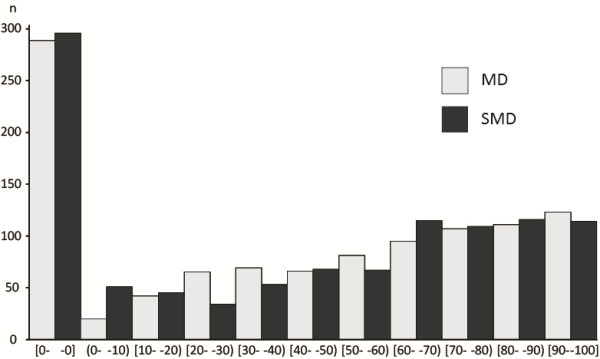
**Histogram of I**^**2 **^**index for MD and SMD.**

Figure 2 shows a histogram containing the I^2^ data. We found, much to our surprise, that the review authors had pooled continuous outcomes as either the MD or the SMD, even when the I^2^ statistics were extremely high. Although the median I^2^ was 47.6% for MD and 48.0% for SMD, in a matched comparison, it was significantly lower for SMD than for MD (*P* < 0.0001, sign test).

### Percentage agreement

In the random-effects model, the median of the percentage agreement in which the point estimate of one trial was included in the 95% confidence interval of the meta-analytically pooled estimate of the remaining trials was 46.7% of the reviews if we used the SMD and 44.5% if we used the MD (*P* < 0.0001, sign test). In the fixed-effect model, the respective figures were 27.8% and 31.9% for the SMD and the MD, respectively (*P* < 0.0001).

The same trend was observed when we recalculated the percentage agreements by simulating a situation where the review authors limited the pooling to moderately heterogeneous (I^2^ index of both MD and SMD, <60%) or to only slightly heterogeneous (I^2^ index of both MD and SMD, <30%) instances (Table 1). In each scenario, the SMD always outperformed the MD in terms of percentage agreement: the random-effects SMD showed the highest agreement of 59.8% when we limited the meta-analyses to cases for which the I-^2^ index of both the MD and the SMD was less than 30%.

**Table 1:** Percentage agreement for the results of a trial to be predicted by the meta-analysed results of the remaining studies in the Cochrane reviews

		**Random-effects model**			**Fixed-effect model**		
	**No. of reviews**	**MD**	**SMD**	***P*-value, sign test**	**MD**	**SMD**	***P*-value, sign test**
All reviews	1068	44.5%	46.7%	<0.0001	27.8%	31.9%	<0.0001
I-squared of both MD and SMD <60%	560	50.0%	52.9%	0.0004	44.0%	47.9%	<0.0001
I-squared of both MD and SMD <30%	357	55.3%	59.8%	<0.0001	53.0%	57.8%	<0.0001

The average percentages are weighted by the number of included RCTs.

#### Z-score

If one of the statistics was statistically more heterogeneous than another, it would be meaningless to discuss their relative detection power. To discuss the statistical power, we compared the Z-score of the MD with that of the SMD using the sign test only when the I^2^ indices of both the MD and the SMD were less than 30%, i.e. less than moderate according to the proposed interpretative guide [1].

In the random-effects model, the median absolute Z-score for the MD was significantly higher than that for the SMD (1.59 vs. 1.61, *P* = 0.007, sign test). In the fixed-effect model, the median Z-score for the MD was again significantly higher than that for the SMD (1.60 vs. 1.63, *P* < 0.0001). However, when the statistical significance was compared at a conventional *P*-value of less than 0.05, no differences in the percentage of “statistically significant” findings were observed between MD or SMD in either the random-effects or the fixed-effect model (*P* = 1.00 and *P* = 0.65, respectively, McNemar test) (Table 2).

**Table 2:** **“Statistically significant” findings for treatment effect in Cochrane reviews only when I**^**2 **^**of both MD and SMD were less than 30%**

	**Random-effects model**	**Fixed-effect model**
	N (%)	N (%)
Both significant	164 (45.9)	168 (47.1)
MD significant but SMD non**-**significant	8 (2.2)	8 (2.2)
MD non**-**significant but SMD significant	11 (3.1)	9 (2.5)
Both non**-**significant	174 (48.7)	172 (48.2)

*P* = 0.647 in random-effects model and *P* = 1.000 in fixed-effect model, according to the McNemar test.

## Discussion

We empirically examined 1068 reviews from the Cochrane Database of Systematic Reviews. The I^2^ index, an index of heterogeneity, was significantly smaller when the meta-analysed results were expressed in terms of the SMD, rather than the MD. When each one of the included RCT was compared against the pooled results of the remaining RCTs in each meta-analysis, the SMD showed a significantly greater percentage agreement than the MD in both the random-effects and the fixed-effect models for all degrees of heterogeneity. On the other hand, no statistically significant difference was found in terms of statistical power for identifying significant results between the MD and the SMD in either the random-effects or the fixed-effect model.

Our research agrees with one previous study regarding heterogeneity and statistical power. This previous study examined a relatively new index of effect, known as the ratio of means, for analysing continuous outcomes in comparison with more traditional effect sizes of continuous outcomes, namely the MD and the SMD. Consequently, the study examined only the meta-analyses where the ratio of means could be calculated, and it further limited the analyses to those meta-analyses containing five or more studies and examined the random-effects model only. However, the study also found that the *P*-value did not statistically differ between the MD and the SMD and that the SMD was less heterogeneous (defined by *P* < 0.1 for the Q statistic) than the MD [17,18]. When we conducted sensitivity analyses using our dataset by including only the meta-analyses that contained five or more studies, the results were also similar.

The percentage agreement figures for continuous outcomes, either in terms of MD or SMD, found in our study appeared to be very low, even when the associated I^2^ index was reasonably low. Clinicians and researchers must therefore be advised to use the most generalizable index of effectiveness, while keeping in mind that the actual degrees of expected overlap may not be as high as one would expect. We found a 5-percentage point difference in the degree of expected agreement when the results were expressed as the SMD vs. when they were presented as the MD; this difference was clinically meaningful and non-negligible.

Much to our surprise, however, the Cochrane authors often pooled their continuous outcomes as the MD and/or the SMD, even when the I^2^ statistics suggested extreme heterogeneity. While it is true that meta-analyses of continuous outcomes tend to be associated with a greater I^2^ value than those of dichotomous outcomes because of the former’s greater statistical power, the generalizability of such meta-analytic results would be highly suspected when the I^2^ values are as high as 80% or 90%.

The comparison of statistical power in the context of greater heterogeneity merits a comment. In this research, we found that the SMD was more generalizable and less heterogeneous than the MD and that there was no significant difference in statistical power between the MD and the SMD. However, strictly and logically speaking, if one statistic is statistically less generalizable and more heterogeneous than another, it would be meaningless to discuss their relative detection power. To discuss statistical power, a high percentage agreement and a low heterogeneity are essential.

As we outlined in the Introduction, whether the MD or the SMD is more clinically preferable as a summary index of meta-analyses of continuous outcomes remains controversial. When the outcome is measured in the same natural unit, such as the amount of bleeding or the number of days of hospitalisation, the MD is definitely better than the SMD from the viewpoint of interpretability. However, our studies have suggested that the SMD may be preferable than the MD from the viewpoint of generalizability. When the outcome is measured using the same patient-reported outcome (PRO) measure, such as the Hamilton Rating Scale for Depression [19], the Short-Form 36 Quality of Life Questionnaire [20], or the Severity Scoring of Atopic Dermatitis [21], even though all the outcomes are measured in the same unit, the superior interpretability of the SMD is not guaranteed unless most clinicians are very familiar with that scale. And for many clinicians in most fields of medicine, such universally known and used PRO instruments are probably rare to non-existent. In such instances, the SMD might be more interpretable than the MD for two reasons. Firstly, the SMD can be interpreted using a general rule of thumb reported by Cohen, in which an SMD of 0.2 represents a small effect, an SMD of 0.5 represents a medium effect, and an SMD of 0.8 or larger represents a large effect [2]. Second, the SMD can be directly and easily converted to a “number needed to treat” if the control event can be assumed [22,23]. In all these instances of PRO instruments, the SMD appears to be more generalizable than the MD.

There is another way to increase interpretability of continuous outcomes. When the minimal important change (MIC) is known, various methods have been proposed to facilitate the interpretability of continuous outcomes [24], including conversion to MIC units and dichotomisation using the MIC threshold. Each has its own advantages and disadvantages, and a comprehensive discussion of their relative merits is out of the realm of the present study. Unfortunately, the MIC is often either not known or, if known, may not be very precise for most of the existing PRO measures.

Finally, SMD may have another important limitation. Because its value derives from the difference in the means between the treatment and control divided by their SD, if variability of the patients is artificially or accidentally reduced, SMD would be overestimated; and if its variability is increased, SMD would be underestimated [25]. However, we think that our results concerning the greater generalizability of SMD have partially resolved such concerns because, despite the variability in the SD across trials, SMD had better external applicability and can therefore be said to have been less vulnerable to over- or underestimation. However, the above mentioned possibility of too small or too large SD, and correspondingly overestimated or underestimated SMD, should always be borne in mind in interpreting SMD.

Our research has two limitations. Firstly, we did not consider the influences of multiple comparisons in our analyses. However, even if we corrected the alpha level from 0.05 to 0.0042 using the Bonferroni method because we made 12 comparisons, only the difference in the absolute Z-score in the random-effects model would lose its significance; all the other results would not change. Secondly we did not categorize the nature of the continuous outcomes. It is possible that subjective continuous outcomes may be more prone to unstable measurement and hence be more heterogeneous than objective continuous outcomes. How generalizability and power may be influenced by such differences in continuous outcomes will be an important research topic in the future.

## Conclusions

When generalizability matters, SMD may be more preferred than MD as a summary measure. In order to increase interpretabillity, SMD can then be supplemented by reporting of MD or some other proposed measures [24].

## Competing interests

The author(s) declare that they have no competing interests.

## Authors’ contributions

NT and TAF conceived and designed the study. NT, YO, AT and YH extracted data. TS checked data extraction and analysed data. All authors commented on drafts of the manuscript and approved the final manuscript.

## Pre-publication history

The pre-publication history for this paper can be accessed here:

http://www.biomedcentral.com/1471-2288/14/30/prepub
